# Ozone‐Induced Rapid and Green Synthesis of Polydopamine Coatings with High Uniformity and Enhanced Stability

**DOI:** 10.1002/advs.202308153

**Published:** 2023-12-19

**Authors:** Liru Tan, Tang Zhu, Yuchan Huang, Huixin Yuan, Ludi Shi, Zijuan Zhu, Pingping Yao, Caizhen Zhu, Jian Xu

**Affiliations:** ^1^ Institute of Low‐dimensional Materials Genome Initiative College of Chemistry and Environmental Engineering Shenzhen University Guangdong 518060 P. R. China

**Keywords:** dopamine, homogeneous coating, ozone, rapid polymerization, surface modification

## Abstract

The development of green, controllable, and simplified pathways for rapid dopamine polymerization holds significant importance in the field of polydopamine (PDA) surface chemistry. In this study, a green strategy is successfully devised to accelerate and control the polymerization of dopamine through the introduction of ozone (O_3_). The findings reveal that ozone serves as an eco‐friendly trigger, significantly accelerating the dopamine polymerization process across a broad pH range, spanning from 4.0 to 10.0. Notably, the deposition rate of PDA coatings on a silicon wafer reaches an impressive value of ≈64.8 nm h^−1^ (pH 8.5), which is 30 times higher than that of traditional air‐assisted PDA and comparable to the fastest reported method. Furthermore, ozone exhibits the ability to accelerate dopamine polymerization even under low temperatures. It also enables control over the inhibition–initiation of the polymerization process by regulating the “ON/OFF” mode of the ozone gas. Moreover, the ozone‐induced PDA coatings demonstrate exceptional characteristics, including high homogeneity, good hydrophilicity, and remarkable chemical and mechanical stability. Additionally, the ozone‐induced PDA coatings can be rapidly and effectively deposited onto a wide range of substrates, particularly those that are adhesion‐resistant, such as polytetrafluoroethylene (PTFE).

## Graphical abstract

A green, controllable and simplified protocol for accelerating dopamine polymerization under a wide range of pH conditions is proposed by utilizing ozone technology. This innovative approach enables the deposition of polydopamine coatings on silicon wafers at an impressive rate of 64.8 nm h^−1^. The resulting polydopamine coatings exhibit a highly homogeneous morphology, good hydrophilicity and exceptional chemical and mechanical stability.

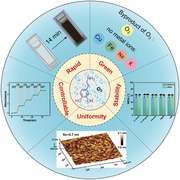

## 1 Introduction

Mussel‐inspired chemistry has garnered significant attention since Messersmith and his co‐workers published their findings on the oxidative polymerization of dopamine and the versatile creation of polydopamine (PDA) coatings on diverse substrates.^[^ ^1^ ^]^ PDA coatings have been extensively studied for surface modification in various applications, such as clean energy batteries,^[^ ^2^ ^]^ healthcare biomedical devices,^[^ ^3^ ^]^ separation membranes,^[^ ^4^ ^]^ and flexible sensors,^[^ ^5^ ^]^ owing to their simplicity, universal adhesion, and post‐functionalization capabilities.^[^ ^6^ ^]^ However, traditional PDA modifications often require alkaline conditions and relatively long reaction time, sometimes exceeding 24 h. This poses challenges for alkaline corrosion and pH sensitive materials. Additionally, controlling the inhibition–initiation of dopamine polymerization has proven to be difficult.^[^ ^7^ ^]^ These limitations restrict the potential applications of polydopamine coatings.

Several approaches have been suggested to accelerate the oxidation and polymerization of dopamine. For instance, Levkin et al. employed UV light irradiation to induce the rapid dopamine polymerization.^[^ ^8^ ^]^ The UV‐irradiated solution exhibited a darker color and higher absorbance at 420 nm compared to the solution under conventional polymerization conditions, indicating that UV irradiation accelerates the rate of dopamine polymerization. Altinkaya et al. proposed a controllable pathway to accelerate the dopamine polymerization using ultrasound as a trigger.^[^ ^9^ ^]^ After 60 min of exposure to ultrasound, the absorbance at 420 nm in the dopamine solution reached 2.31, significantly higher than the value observed in conventional polymerization. Apart from physical approaches, the addition of metal ions, strong chemical oxidants, or biocatalysts has emerged as a highly effective approach for accelerating dopamine polymerization in surface coating applications. Zhao et al. demonstrated that polydopamine coatings can be prepared in acidic or neutral aqueous solutions through ammonium persulfate‐induced polymerization, thereby expanding the application range of dopamine surface modification technology in materials.^[^ ^10^ ^]^ Furthermore, the CuSO_4_/H_2_O_2_ hybrid system can trigger rapid polymerization and uniform nucleation of dopamine, allowing for the deposition of PDA nanoparticles on diverse substrates. It is worth noting that dopamine polymerization is not accelerated by H_2_O_2_ alone but is significantly sped up by copper ions.^[^ ^11^ ^]^ Other oxidants, including sodium periodate,^[^ ^12^ ^]^ potassium permanganate,^[^ ^13^ ^]^ laccase,^[^ ^14^ ^]^ and horseradish peroxidase,^[^ ^15^ ^]^ have been proposed to significantly accelerate dopamine polymerization. However, these current oxidants exhibit critical limitations, such as being difficult to tune, requiring large dosages, being expensive, and causing secondary pollution. Therefore, there is an urgent need to develop a controllable, inexpensive, readily available, and environmentally friendly strategy to accelerate dopamine polymerization and PDA deposition.

Ozone (O_3_) possesses a high redox oxidation potential of 2.07 V. As a strong oxidant, it finds application in various areas such as the purification of surface or groundwater for potabilization, as well as in wastewater treatment for the elimination of microorganisms, inorganic ions, and organic pollutants.^[^ ^16^ ^]^ Herein, we present a highly efficient, eco‐friendly, and cost‐effective approach to accelerate dopamine polymerization and PDA coatings deposition on diverse surfaces through the introduction of ozone. In an aqueous solution, ozone can generate a multitude of reactive oxygen species (ROS), including hydroxyl (•OH), superoxide (O_2_•^−^), and ozonide (O_3_•^−^) radicals.^[^ ^16^, ^17^ ^]^ These ROS play a crucial role in the rapid oxidation and polymerization of dopamine and the deposition of PDA coatings on material surfaces. The most advantageous aspect of the ozone‐induced system, compared to traditional methods, is that the decomposition product of ozone is oxygen and no additional metal ions are required during the reaction process, ensuring the absence of secondary pollution. This system exhibits environmentally friendly, cost‐effective, and time‐saving features.

## 2 Results and Discussion

**Figure 1:**
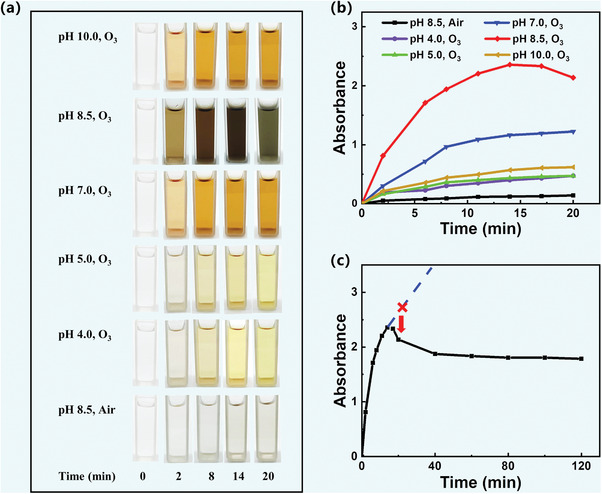
a) Photographs and b) time‐dependence of absorbance at 420 nm of diluted dopamine solutions with different pH and additives at different time points. c) Time‐dependence of absorbance at 420 nm for diluted dopamine solution (pH 8.5) containing ozone. UV–vis absorbance and photographs of dopamine solutions were measured after being diluted fivefold.

To demonstrate that ozone could induce the polymerization of dopamine, ozone gas was continuously bubbled into a dopamine solution (2 mg mL^−1^, Tris‐HCl buffer, pH 8.5) at a flow rate of 5 g h^−1^. **Figure** **1**a depicts the color variations of the dopamine solution as it undergoes continuous bubbling with ozone gas at different reaction times. In just 2 min, the dopamine solution infused with ozone (pH 8.5) undergoes a rapid darkening process, transitioning to a dark brown coloration within 8 min, and nearly black after 14 min. In contrast, the dopamine solution under air (pH 8.5) only exhibits a light grey color after 20 min. The polymerization process was further confirmed through UV–vis spectra. As shown in Figure S1 (Supporting Information), a characteristic peak at 420 nm emerges, indicating the polymerization of dopamine. Moreover, the peak intensity at 420 nm gradually increases over time, reaching a value of 2.38 after bubbling ozone for 14 min (Figure 1b). In contrast, the UV–vis absorption intensity of the dopamine solution after a 14 min reaction under ambient air conditions at 420 nm is merely 0.14 (Figure 1b). These results clearly demonstrate that ozone can induce and significantly accelerate the dopamine polymerization process. This acceleration can be attributed to the high redox oxidation potential of ozone (2.07 V), and the reactive oxygen species generated from ozone decomposition, including hydroxyl (•OH), superoxide (O_2_•^−^), and ozonide (O_3_•^−^) radicals.^[^ ^16^, ^17^ ^]^ These ROS play a key role in the rapid oxidation and polymerization of dopamine.

Typically, dopamine polymerization in the presence of air requires weak alkaline conditions (pH 8.5). However, ozone can facilitate dopamine polymerization over a broader pH range. Figure 1a shows the distinct color changes in the ozone‐induced dopamine oxidation reaction under different pH values. At pH 4.0 and pH 5.0, the solution turns light yellow within 20 min. At pH 7.0 and pH 10.0, the dopamine solution transforms from clear and transparent to dark brown. Compared to air‐assited dopamine polymerization, ozone‐induced dopamine polymerization is accelerated in weak acidic, neutral, and alkaline aqueous solutions. Furthermore, within the pH range of 4.0 to 10.0, the peak intensity of the UV absorption curve at 420 nm for the ozone‐induced dopamine solution is significantly higher than that of the dopamine solution reacted under air for the same duration (Figure 1b; Figure S2, Supporting Information). These findings provide strong evidence that ozone effectively accelerates dopamine polymerization across a broader pH range, spanning from pH 4.0 to 10.0. Additionally, in the same reaction time, the absorption of the ozone‐induced dopamine solution at 420 nm under pH 8.5 exhibits the highest value (Figure 1b), further confirming that the rate of dopamine polymerization in a weak alkaline buffer system is faster than in neutral, acidic and strong alkaline systems.

Interestingly, with ozone treatment time exceeding 17 min, the color of the dopamine solution gradually lightens. At the same time, the peak intensity at 420 nm of the ozone‐induced dopamine solution decreases after 14 min and remains constant at 1.79 from 40 to 120 min (Figure 1c; Figures S1b and S3, Supporting Information). This change primarily arises from the decomposition effect of ozone, leading to a reduction in the concentration of polydopamine. Moreover, beyond 120 min, the color of the dopamine solution continues to lighten, eventually reaching a nearly colorless state by 480 min (Figure S3, Supporting Information). This indicates that during the later stage of the reaction, the decomposition reaction of PDA becomes predominant over dopamine polymerization, leading to a gradual decrease in the concentration of PDA in the solution. This phenomenon highlights the self‐cleaning nature of our proposed approach and its significant potential to mitigate environmental pollution. The experimental findings mentioned above demonstrate that introducing ozone gas into a dopamine solution at pH 8.5 for a duration of 14 min yields the highest concentration of polydopamine.

**Figure 2:**
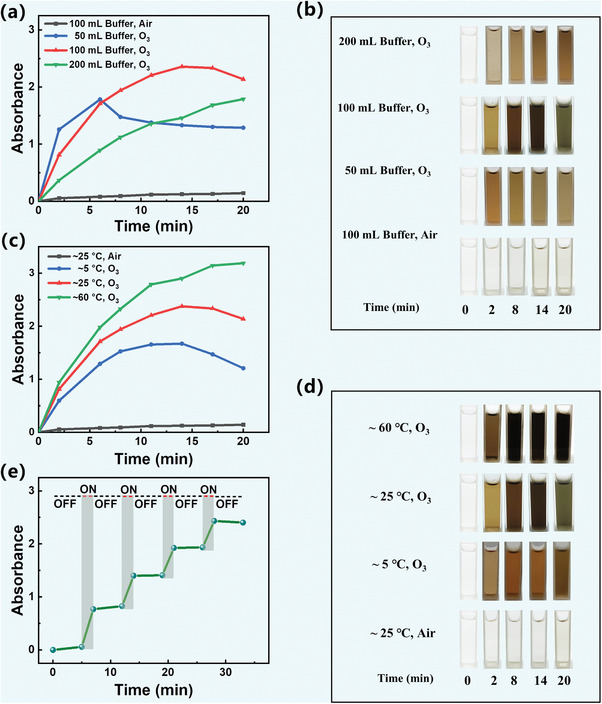
a) Time‐dependent of absorbance at 420 nm and b) photographs of dopamine solution (pH 8.5) with different ozone concentrations. c) Time‐dependent of absorbance at 420 nm and d) photographs of dopamine solution (pH 8.5) at different temperatures. e) Absorbance changes by turning on (2 min) and off (5 min) the ozone at pH 8.5. UV–vis absorbance and photographs of dopamine solutions were measured after being diluted fivefold.

The impact of ozone concentration on the rate of dopamine polymerization was investigated. The experiments were conducted by adjusting the volume of the buffer solution while the dopamine concentration remained at 2 mg mL^−1^, and the ozone flow rate was maintained at 5 g h^−1^. The experimental data reveals that increasing the volume of the buffer solution from 100 to 200 mL (resulting in a halved ozone concentration) leads to a significant decrease in the UV–vis absorption intensity of the dopamine solution at 420 nm (**Figure** **2**a). Furthermore, the color transformation of the dopamine solution exhibits a decelerated progression, shifting from colorless to brown over a span of 20 min (Figure 2b). These results indicate a noteworthy reduction in the rate of dopamine polymerization associated with a lower ozone concentration. Conversely, when the volume of the buffer solution is reduced from 100 to 50 mL (resulting in a doubled ozone concentration), the UV–vis absorption intensity of the dopamine solution at 420 nm initially exhibits a substantial increase. For a duration of 6 min, the UV–vis absorption intensity reaches its peak and subsequently displays a declining trend (Figure 2a). Additionally, the color of the dopamine solution rapidly darkens within a few minutes and then gradually lightens (Figure 2b). This observation suggests that the excessively high ozone concentration may have caused the degradation of PDA in the solution.

Subsequently, the influence of temperature on the rate of dopamine polymerization was also explored. Specifically, ozone‐induced dopamine polymerization was examined at lower temperature (≈5 °C), room temperature (≈25 °C) and higher temperature (≈60 °C), all with the same ozone gas rate and dopamine concentration. Temperature exerts a multifaceted impact on ozone‐induced dopamine polymerization. On the one hand, temperature affects the solubility of ozone, with higher temperatures resulting in lower ozone solubility. On the other hand, increasing temperature can elevate collision frequency and enhance the reaction rate, thereby accelerating dopamine polymerization.^[^ ^18^ ^]^ The experimental results demonstrate that, under identical reaction times, higher temperatures correspond to greater UV–vis absorption at 420 nm and darker color of the solution (Figure 2c,d;Figure S5, Supporting Information). This finding indicates that elevating the temperature can expedite dopamine polymerization in the ozone‐induced dopamine polymerization system. Furthermore, even under low temperature (≈5 °C), the rate of ozone‐induced dopamine polymerization remain significantly higher than air‐assisted dopamine polymerization at room temperature. This suggests that the ozone‐induced dopamine polymerization has the potential to expand the range of dopamine modification, particularly for materials that require low‐temperature reactions.

To demonstrate the effectiveness and controllability of the ozone‐induced dopamine polymerization, the solution (pH 8.5) was bubbled with ozone gas for 2 min (ON) and then to the conventional polymerization for 5 min (OFF), and this cycle was repeated four times. The results in Figure 2e and Figure S6 (Supporting Information)show that the absorbance intensity of the dopamine solution at 420 nm increased sharply when ozone was introduced. On the contrary, the change was insignificant during the conventional polymerization. This phenomenon can be attributed to the high reactivity and short half‐life of the ozone generated ROS.^[^ ^8^ ^]^ Consequently, the polymerization of dopamine can be conveniently controlled by properly regulating the “ON/OFF” mode of ozone gas.

**Figure 3:**
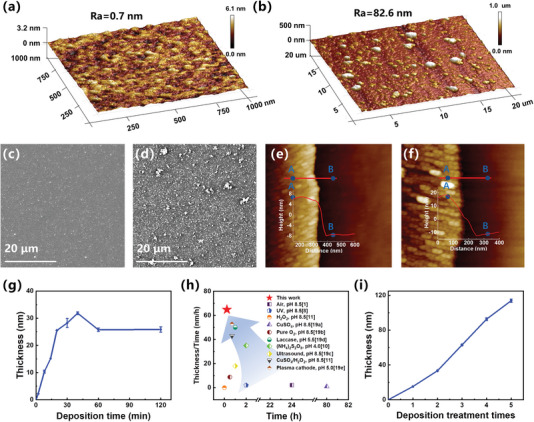
a) AFM and c) SEM images of PDA‐coated silicon wafer induced by ozone for 14 min. b) AFM and d) SEM images of PDA‐coated silicon wafer assisted by air for 24 h. AFM measurement of the ozone‐induced PDA coatings thickness at different time via a scratch test: e) 14 min, and f) 30 min. g) Time‐dependence of thickness for the ozone‐induced PDA coatings deposited on silicon wafer, which determined by ellipsometry. h) Comparisons of deposition rates for PDA coatings using various methods with a dopamine concentration of 2 mg mL^−1^. i) Thickness of ozone‐induced PDA coatings deposited on silicon wafer for different times.

Surface morphology and deposition rate are crucial factors in PDA coatings, thus, the polymerization of dopamine on a silicon wafer was investigated. The 3D phase AFM images demonstrate the ozone‐induced PDA coatings have a homogeneous morphology, with an average roughness (Ra) of 0.7 nm (**Figure** **3**a). In contrast, the air‐assisted PDA coatings exhibit particle aggregates and a larger Ra of 82.6 nm (Figure 3b). Additionally, the SEM images reveal that the ozone‐induced PDA coatings exhibit a significantly more uniform surface compared to the air‐assisted PDA coatings (Figure 3c,d). These findings strongly indicate that under ozone conditions, the deposition of PDA results in a remarkably uniform surface coating, especially when compared to PDA deposition under ambient conditions. The thickness of the PDA coatings was evaluated by using both AFM assessment via a scratch test and ellipsometry. The AFM results indicate that after bubbling ozone gas for 14 and 30 min, the thickness of the PDA coatings was measured to be 15.12 and 28.23 nm, respectively (Figure 3e,f). Similarly, the ellipsometry data show a gradual increase in the thickness of the PDA coatings over time. At 14 min, the ozone‐induced PDA coatings reach a thickness of 15 nm, while at 30 min, it reaches a thickness of 31 nm (Figure 3g). These findings from ellipsometry are consistent with the AFM measurements, providing further confirmation of the coatings thickness. Consequently, the deposition speed of ozone‐induced PDA reaches ≈64.8 nm h^−1^, which is 30 times higher than that of traditional air‐assisted PDA and comparable to the fastest reported method (Figure 3h).^[^ ^1^, ^8^, ^10^, ^11^, ^19^ ^]^ What is noteworthy is that as the deposition time is extended, the thickness of the PDA coatings exhibits a slight decrease, eventually stabilizing at 25.84 nm between 60 and 120 min (Figure 3g). This finding suggests that ozone affects polydopamine degradation both in aqueous media and on the substrate surface. However, the decomposition of the PDA coatings on the substrate is limited due to its dense nature, making it difficult for ROS to penetrate the coatings. Additionally, increasing the number of reactions can enhance the thickness of the ozone‐induced PDA coatings. Figure 3i illustrates a linear relationship between the ozone‐induced PDA coatings thickness and treatment times. After five deposition cycles in dopamine solution (pH 8.5) infused with ozone for 14 min, the thickness of the PDA coatings reached 110.5 nm. Interestingly, the thickness of the PDA coatings in the latter three deposition cycles exceeds that of the initial two cycles. This observation can be attributed to the progressive coverage of the silicon wafer surface by PDA as the number of depositions increases, indicating a transition from silicon as the substrate to PDA itself. The presence of abundant hydroxyl, amino, quinone, benzene ring, and indole groups within PDA molecules facilitates the formation of a greater number of strong interactions among PDA molecules. These strong interactions may contribute to the formation of thicker PDA coatings.^[^ ^20^ ^]^

**Figure 4:**
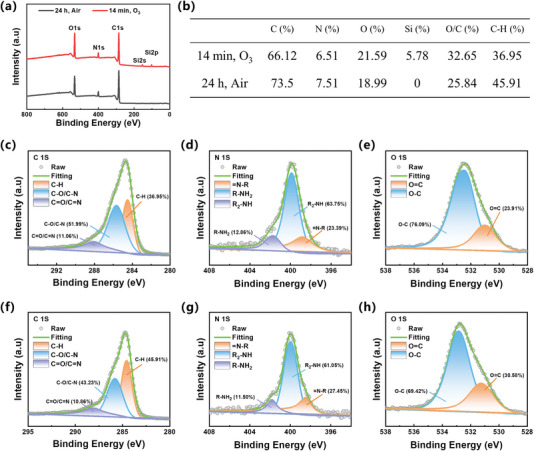
a) XPS spectra of ozone‐induced and air‐assisted PDA coatings. b) Elemental compositions (in atomic percentage) and C 1s core‐level components of both PDA coatings examined by XPS. High‐resolution c) C 1s, d) N 1s and e) O 1s XPS spectra of ozone‐induced PDA coatings. High‐resolution f) C 1s, g) N 1s and h) O 1s XPS spectra of air‐assisted PDA coatings.

XPS analysis was performed to examine the elemental composition of the ozone‐induced PDA coatings and air‐assisted PDA coatings. The ozone‐induced PDA coatings were obtained by immersing a Si substrate in a dopamine solution while continuously bubbling ozone gas for 14 min. On the other hand, the air‐assisted PDA coatings were obtained by immersing a Si substrate in a dopamine solution under ambient air conditions for 24 h. The ozone‐induced PDA coatings have relatively higher atomic percentages of oxygen (O) and the O/C ratio compared to the air‐assisted PDA coatings, indicating the formation of more carbonyl groups or quinoid structures (**Figure** **4**a,b). Furthermore, the C 1s spectra of both PDA coatings can be fitted into three component peaks. The signal at 284.5 eV was assigned to carbon in C─H bonds, the signal at 285.7 eV was attributed to carbon in ether bonds (C─O) and amino groups (C─NH_2_), and the signal at 288.1 eV was associated with carbon in C═N and C═O bonds (Figure 4c,f).^[^ ^21^ ^]^ Notably, the ozone‐induced PDA coatings have a lower ratio of C─H (36.95%) compared to the air‐assisted PDA coatings (45.91%) (Figure 4b,c,f). The reduction in the C‐H ratio may be attributed to both the cyclization and covalent connection of dopamine quinone, which generate numerous covalent linkages.^[^ ^11^ ^]^ Moreover, the N 1s signals of both PDA coatings were resolved into three distinct component peaks representing R‐NH_2_ (B.E. = 401.9 eV), R_2_NH (B.E. = 399.9 eV), and = NR (B.E. = 398.7 eV), demonstrating the cyclization of dopamine quinone (Figure 4d,g). The O 1s spectra in Figure 4e,h were resolved into two individual component peaks at 532.7 and 531.1 eV assigned to oxygen from catechol of dopamine (C─O) and quinone (C═O), which indicates the oxidation of phenol to quinone structure.^[^ ^21^ ^]^

**Figure 5:**
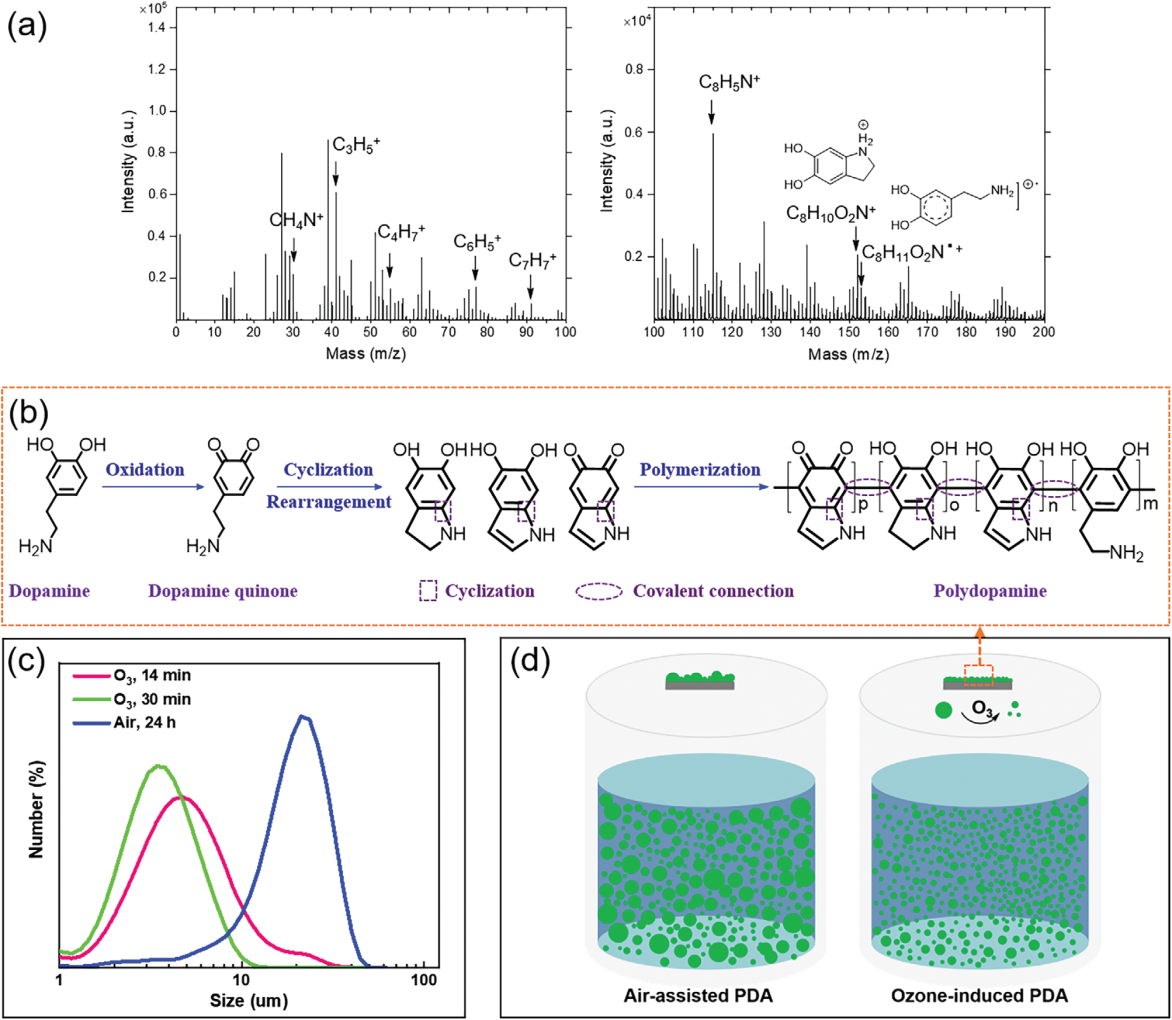
a) Positive ToF‐SIMS ion spectra of ozone‐induced PDA coating. b) Process of the ozone‐induced dopamine polymerization. c) The particle size and distribution of the PDA in dopamine solution after reaction under air for 24 h or under ozone for 14 and 30 min. d) Schematic illustration depicting the formation of PDA from a dopamine solution under ambient air or ozone conditions.

The positive time‐of‐flight secondary ion mass spectrometry (ToF‐SIMS) spectrum of ozone‐induced PDA exhibited several peaks at m/z 30 (CH_4_N^+^), 41 (C_3_H_5_^+^), 55 (C_4_H_7_^+^), 77 (C_6_H_5_^+^), 91 (C_7_H_7_^+^), 115 (C_8_H_5_N^+^), 152 (C_8_H_10_O_2_N^+^), 153 (C_8_H_11_O_2_N^·+^) (**Figure** **5**a). These findings offer proof of the existence of both uncyclized (catecholamine) and cyclized (indole) units in the ozone‐induced PDA coating.^[^ ^22^ ^]^ Furthermore, the ozone‐induced PDA exhibits a smaller average particle size compared to the air‐assisted PDA. Notably, the average particle size of PDA formed under ozone after a 30 min reaction is smaller than that formed after a 14 min reaction, indicating that ozone‐induced decomposition of PDA in the solution leads to a more uniform distribution of PDA particles (Figure 5c). Based on these results, a mechanism for the ozone‐induced dopamine polymerization was proposed. In the dopamine polymerization system involving ozone, the abundant free radicals generated by ozone accelerate dopamine oxidation, promote intramolecular cyclization, and facilitate intermolecular covalent bond formation. Additionally, the substantial production of oxygen resulting from ozone decomposition promotes the formation of highly conjugated and planar structures, thereby facilitating enhanced molecular stacking through mechanisms such as charge transfer, hydrogen bonding, and π–π stacking interactions.^[^ ^19b^ ^]^ Moreover, the decomposition effect of ozone on PDA prevents the formation of large particle sizes, resulting in a relatively uniform distribution of PDA particles in solution (Figure 5b,d). As a result, the ozone‐induced PDA exhibits a more homogeneous surface morphology.

**Figure 6:**
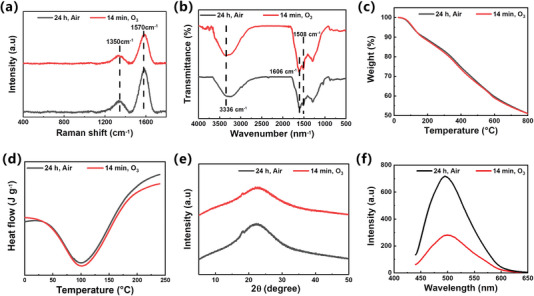
a) Raman, b) ATR‐FTIR, c) TG, d) DSC, e) XRD and f) Fluorescence spectra of air‐assisted and ozone‐induced PDA coatings.

Raman spectra analysis reveals the presence of two distinct peaks at ≈1350 and 1570 cm^−1^ in both ozone‐induced and air‐assisted PDA coatings (**Figure** **6**a). These peaks provide evidence of the graphite‐like structures inherent in PDA.^[^ ^23^ ^]^ ATR‐FTIR spectra demonstrate a broad band at 3336 cm^−1^, assigned to N─H and O─H stretching of PDA. Additionally, the absorption peaks at 1508 and 1606 cm^−1^ correspond to the primary amine N─H bending and C═C aromatic stretching of indole or indoline structures, respectively (Figure 6b).^[^ ^21^, ^24^ ^]^ These data align with the spectra of air‐assisted PDA. Thermogravimetric analysis of both ozone‐induced and air‐assisted PDA coatings demonstrates similar and multi‐stage weight reduction, indicating chemical heterogeneity (Figure 6c). The thermal degradation of PDA exhibits four distinct steps. The initial step, observed from 25 to 100 °C, corresponds to the evaporation of loosely bound water caused by the high hygroscopic nature of PDA. The second step, occurring from 100 to 290 °C, involves the liberation of tightly bound intramolecular water. The third step, from 290 to 590 °C, is attributed to the decomposition of aliphatic components originating from non‐oxidized dopamine during polymerization. Finally, the last step, from 598 to 800 °C, corresponds to the decomposition of aromatic compounds such as catechol or o‐benzoquinone moieties.^[^ ^25^ ^]^ The final average mass retention of PDA is 51.0%. DSC analysis of PDA reveals an endothermic peak at 100 °C, corresponding to the melting process of PDA (Figure 6d). The ozone‐induced PDA exhibits an equivalent melting enthalpy to the air‐assisted PDA. XRD spectra show the same diffraction peak observed at 23.2 ± 0.1° (2*θ*) for both ozone‐induced and air‐assisted PDA coatings (Figure 6e), indicating a similar crystalline structure. Additionally, Figure 6f illustrates that both ozone‐induced and air‐assisted polydopamine solutions exhibit a prominent fluorescence emission peak at 510 nm.^[^ ^26^ ^]^ Although the complete understanding of PDA structures is still being explored, the aforementioned results illustrate that the ozone‐induced PDA coatings exhibit similar crystallization behavior, thermal properties, and fluorescence characteristics to the air‐assisted PDA coatings.

**Figure 7:**
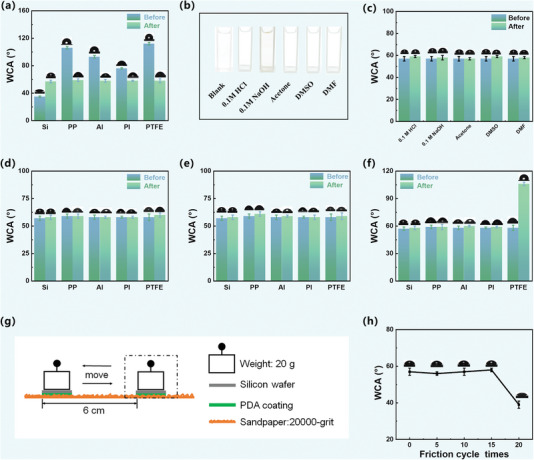
a) WCAs of different substrates before and after being coated with ozone‐induced PDA for a duration of 14 min. b) Digital photographs of the eluents in the chemical stability tests. c) WCAs of the ozone‐induced PDA‐coated silicon wafers before and after being immersed in different solvents for 2 h. WCAs of the ozone‐induced PDA‐coated substrates before and after ultrasonic treatment in d) ultrapure water or e) ethanol. f) WCAs of the ozone‐induced PDA coated substrates before and after tape experiment. g) Schematic diagram of the friction test, and h) WCAs of the PDA coated Si substrate before and after different cycles of friction test.

The deposition of PDA is versatile and enables hydrophilic modification of various substrate surfaces. In addition to silicon wafers, PDA coatings can be rapidly deposited on several substrates by simply immersing them into a dopamine solution (pH 8.5) infused with ozone for 14 min. The initial water contact angles (WCAs) of aluminum (Al), polypropylene (PP) membranes, polyimide (PI) membranes, and polytetrafluoroethylene (PTFE) are measured to be 93 ± 1°, 106 ± 2°, 76 ± 1°, 112 ± 2°, respectively. However, after the deposition of ozone‐induced PDA coatings, the WCAs of all substrates change to ≈57°, which approaches the theoretical value of pure PDA coatings (**Figure** **7**a). The enhanced hydrophilicity of the substrates after PDA modification can be attributed to the presence of various functional groups in PDA, including carbonyl, amino, imine, and phenol groups. These results indicate the material‐independent nature of the PDA coatings, especially for those adhesion‐resistant materials like PTFE.

The stability of the PDA coatings is a crucial factor for its applications in material‐modified surfaces. To evaluate the chemical stability of the PDA coatings, ozone‐induced PDA‐coated silicon wafers (14 min) were immersed in various solutions for 2 h, including strong acid (0.1 m HCl), strong alkali (0.1 m naoh), acetone, N,N‐dimethylformamide (DMF), and dimethyl sulfoxide (DMSO). It is observed that the eluent turns light grey (Figure 7b) and the UV–vis spectrum shows minimal absorption at 420 nm for the strong alkali (0.1 m NaOH) treatment (Figure S7b, Supporting Information), indicating a small amount of PDA decomposed. This is consistent with previous findings that PDA undergoes inherent decomposition in strong alkaline conditions.^[^ ^27^ ^]^ However, the eluents for the other solutions remain colorless and transparent (Figure 7b), and their UV–vis spectra exhibit no significant absorption at 420 nm (Figure S7, Supporting Information). Furthermore, the WCAs of ozone‐induced PDA‐coated silicon wafers show little noticeable change before and after treatment, even for the sample immersed in strong alkali (Figure 7c). These results suggest that ozone‐induced PDA coatings exhibit good chemical stability.

To investigate the mechanical stability of the ozone‐induced PDA coatings, a PDA‐coated silicon wafer was subjected to ultrasonic treatment in ultrapure water or ethanol for three cycles of 10 min each. The WCAs remain unchanged after the treatment, and the eluents remain colorless and transparent (Figure 7d,e; Figure S8, Supporting Information). Similar results were observed for other PDA‐coated substrates, including PP, Al, PI, and PTFE, where the WCAs show no significant changes after undergoing the same ultrasonic treatment in ultrapure water or ethanol (Figure 7d,e). These findings confirm that ozone‐induced PDA coatings exhibit remarkable mechanical stability on various substrates. The exceptional stability of ozone‐induced PDA coatings can be attributed to the formation of more chemical bonds and a more uniform and smooth morphology, as verified by SEM, AFM, XPS, and ToF‐SIMS analysis (Figures 3, 4, 5).

In the tape experiments, the WCAs of samples with PDA deposited on silicon wafer, aluminum sheet, PP film, and PI film showed negligible variation, measuring 58 ± 2°, 60 ± 1°, 59 ± 3°, and 59 ± 1°, respectively, after the tape experiments. However, in the case of PDA‐coated PTFE, the PDA coating was predominantly transferred to the tape surface upon tape adhesion, resulting in a WCA of 106 ± 2° (Figure 7f). Additionally, tape experiments were conducted on air‐assisted PDA‐coated PTFE, revealing significant removal of the PDA coating by the tape and resulting in a WCA of 95 ± 2° (Figure S9, Supporting Information). These results indicate that the PDA coatings exhibit robust adhesion to the surfaces of silicon wafer, aluminum sheet, PP film, and PI film during tape experiments. However, further improvements are necessary to enhance the adhesion of both air‐assisted and ozone‐induced PDA coatings on PTFE surfaces. In the friction tests, a PDA‐coated silicon wafer was placed face‐down onto a 20 000‐grit sandpaper, with a 20 g weight applied on top of the substrate. A reciprocating motion was applied horizontally, covering an abrasion length of 6 cm (Figure 7g). After 5, 10, 15, and 20 cycles of friction testing, the WCAs of the coating were measured as 57 ± 2°, 56 ± 1°, 58 ± 1°, and 39 ± 2°, respectively (Figure 7h). These findings demonstrate that the ozone‐induced PDA coating on the silicon wafer exhibits a certain degree of wear resistance, remaining stable up to 15 cycles of friction. However, beyond 15 cycles, the coating experiences damage, leading to substrate exposure and a significant change in the surface contact angle.

## 3 Conclusion

In conclusion, a novel protocol for accelerating dopamine polymerization using ozone under acidic, neutral, and basic conditions has been presented. The attractive feature of this approach is that ozone significantly reduces the hydrophilic modification time to as short as 14 min, while achieving a remarkable deposition rate of PDA coatings on silicon wafers at 64.8 nm h^−1^, which is comparable to the fastest existing methods. The high reactivity and short half‐life of ozone‐generated reactive oxygen species allow convenient control of dopamine polymerization by regulating the “ON/OFF” mode of ozone gas. Furthermore, this approach exhibits noticeable acceleration even at lower temperatures, indicating its great potential for applications in low‐temperature modification scenarios. Moreover, the ozone‐induced dopamine polymerization can be applied to coat various surfaces, including inorganic materials (such as Si), metals (such as Al), and plastics (such as PP, PI, or PTFE). The resulting PDA coatings demonstrate a homogeneous morphology, good hydrophilicity, and excellent chemical and mechanical stability. Importantly, this synthesis method is green and clean, as ozone decomposes into oxygen as a byproduct, and no additional metal ions are required during the reaction process, thus avoiding potential impurities and contamination in the resulting PDA coatings.

## 4 Experimental Section

### Materials

Dopamine hydrochloride and tris (hydroxymethyl) aminomenthane (Tris) were obtained from Shanghai Macklin Biochemical Co., Ltd. (China). Silicon (Si) wafer (2 cm × 2 cm), aluminum (Al) sheets, polypropylene (PP) membranes, polyimide (PI) membrane, and polytetrafluoroethylene (PTFE) are commercial products. Prior to use, silicon wafers were subjected to a 20 min immersion in boiling piranha solution (3:7 v/v H_2_O_2_–H_2_SO_4_), followed by thorough rinsing with deionized water and drying with nitrogen gas. Other substrates were cut into squares with a side length of 2 cm, washed by acetone overnight to remove impurities, and then dried in a vacuum oven at 30 °C. Reagents such as sodium hydroxide, sulphuric acid (98%), hydrogen peroxide (30%), ethanol, hydrochloric acid, acetone, dimethyl sulfoxide (DMSO) and N,N‐dimethylformamide (DMF) were obtained from Shanghai Macklin Biochemical Co., Ltd. and used without further purification. Ozone was generated electrically from air source using an ozone generator (220 V/50 Hz, HY‐004‐5A, Guangzhou Jiahuan electrical technology Co., Ltd., China). Different pH buffer solutions were purchased from Quanzhou Yida technology Co., Ltd. (China).

### Ozone‐Induced Dopamine Polymerization

200 mg of dopamine hydrochloride was dissolved in 100 mL of buffer solution (2 mg mL^−1^) with pH values ranging from 4.0 to 10.0. Simultaneously, ozone gas was introduced into the dopamine solution by using an ozone generator at a flow rate of 5 g h^−1^. The color changes and UV–vis spectra of the dopamine solution were observed at specific time intervals after being diluted fivefold.

### Ozone‐Induced Dopamine Polymerization at Different Temperatures

First, the Tris‐HCl buffer solution (pH 8.5) was equilibrated in a cold bath or oil bath to reach the desired temperature, such as 5 or 60°C, ensuring thermal stabilization. Subsequently, dopamine (2 mg mL^−1^) was introduced into the buffer solution, followed by the prompt introduction of ozone to expedite the rapid polymerization of dopamine. The UV–vis absorption spectra of the solution were measured at various reaction times after undergoing a fivefold dilution.

### ON‐OFF Experiment

The dopamine solution (2 mg mL^−1^) was first incubated in Tris‐HCl buffer (pH 8.5) for 5 min before recording the UV–vis absorption spectra. Subsequently, ozone gas was bubbled into the solution for 2 min, and then UV–vis absorbance was immediately measured. This on‐off cycle was repeated four times, and the resulting change in absorbance at 420 nm was calculated.

### Air‐Assisted Dopamine Polymerization

As a control experiment, dopamine was dissolved in a Tris‐HCl buffer solution (pH 8.5) with a concentration of 2 mg mL^−1^. The reaction took place in the presence of air, with the container left open and stirring at a speed of 400 rpm. The color changes and UV–vis spectra of the dopamine solution were observed at specific time intervals after being diluted fivefold.

### Deposition of PDA Coatings on Various Substrates Induced by Ozone

The pre‐cleaned silicon wafers were soaked in a freshly prepared solution of dopamine (2 mg mL^−1^) in Tris‐HCl buffer solution (pH 8.5). Subsequently, ozone gas was introduced into the solution at a flow rate of 5 g h^−1^. After a predetermined period of time, the wafers were taken out, washed thoroughly with water, and dried under vacuum conditions at 30 °C for 4 h. The same method was applied to other substrates. Unless otherwise specified, the ozone‐induced PDA coatings on the substrates were prepared by continuously introducing ozone into the dopamine Tris‐buffer solution (pH 8.5) for 14 min. For comparison, the air‐assisted PDA coatings on the silicon wafer were obtained by continuously stirring the dopamine Tris‐buffer solution (pH 8.5) under ambient air conditions for 24 h.

### Stability Test of the PDA Coatings

The chemical stability of the PDA coatings was assessed by immersing PDA‐modified Si substrates in various solvents, including 0.1 m HCl, 0.1 m NaOH, acetone, DMSO, and DMF, for a duration of 2 h. Additionally, the mechanical stability of the PDA coatings was evaluated through sonication treatment, tape experiments and friction tests. In the sonication tests, the PDA‐modified substrates underwent three rounds of 10 min sonication in either water or ethanol. Subsequently, the water contact angle (WCA) of the dried PDA‐coated substrates and the UV–vis absorption spectra of the eluents were measured. For the tape experiments, 3 M Scotch Transparent Tape 600, with a peel strength of 0.44 N mm^−1^ was applied to various PDA‐coated substrates. After a static period of 2 min, the tape was rapidly peeled off at a 90° angle perpendicular to the surface. The changes in WCAs on the surfaces before and after tape removal were then compared. In the friction tests, a PDA‐coated silicon wafer was placed face‐down onto a 20 000‐grit sandpaper, with a 20 g weight applied on top of the substrate. A reciprocating motion was applied horizontally, covering an abrasion length of 6 cm. After different cycles of testing, the WCAs were measured.

### Characterization

The UV–vis spectra of the solution were measured at predetermined time intervals by using a UV–vis spectrometer (UV‐2600, Shimadzu, Japan). Surface morphology of the samples was observed by using scanning electron microscopy (SEM, S‐3400N, Hitachi, Japan) and atomic force microscopy (AFM, Dimension Icon, Bruker, Germany). The thickness of PDA coatings was determined by using AFM via a scratch test and spectroscopic ellipsometry (RC2 Xl+, J. A. Woollam, USA). Ellipsometry measurements were performed at an incident angel of 70°, with the wavelength ranging from 370 to 1000 nm at five different locations on each sample, and the average thickness was calculated. Fourier transform infrared (FTIR) spectra were obtained using a FTIR spectrophotometer (Tensor 27, Bruker, Germany). X‐ray photoelectron spectra (XPS) were collected by using a spectrometer from Thermo Fisher Technologies (Czech) with Al Kα excitation radiation (1486.6 eV). X‐ray diffraction (XRD) patterns were recorded by using an X‐ray powder diffractometer (Rigaku, Miniflex‐600, Germany) with Cu Kα radiation within the range from 5 to 50°. Raman spectra (Invia, Renishaw, UK) were used to determine the composition of the polydopamine coatings. A fluorescence spectrophotometer (F7000, Hitachi, Japan) was employed to record the fluorescence spectrum of dopamine solution. The specific parameters included an excitation wavelength of 440 nm and an emission wavelength ranging from 440 to 830 nm. Thermal transitions were obtained by using Differential Scanning Calorimetry (DSC 3‐STAR System, Mettler toledo, USA) with a heating rate of 10 °C min^−1^ from −50 to 250 °C under a nitrogen atmosphere. Thermogravimetric Analysis (TGA 2‐STAR System, Mettler toledo, USA) was performed with a heating rate of 10 °C min^−1^ from room temperature to 800 °C under a nitrogen atmosphere. The water contact angle (WCA) was measured using a drop shape analysis instrument (SDC‐350, SINDIN, China) at ambient temperature via a sessile drop method. A water droplet of 5 µL was used and each contact angle value was an average of five measurements taken at different positions on the surface. The time‐of‐flight secondary ion mass spectrometry (ToF‐SIMS) spectrum of ozone‐induced PDA coating was obtained using a ToF‐SIMS V spectrometer (ION‐TOF GmbH, Munster, Germany). The Freshly prepared ozone‐induced PDA coating was bombarded with Bi^3+^ primary ions accelerated at 30 kV. The raster area measured 100 µm × 100 µm, and each spectrum was acquired for 40 s. The particle size and distribution of PDA formed in the dopamine solution after reaction under air or ozone conditions were analyzed using a BT‐9300ST laser particle size analyzer (Baite Instrument Co., Ltd., China).

## Conflict of Interest

The authors declare no conflict of interest.

## Supporting information

Supporting Information

## Data Availability

The data that support the findings of this study are available from the corresponding author upon reasonable request.
