# Supplementary material for: Ozone‐Induced Rapid and Green Synthesis of Polydopamine Coatings with High Uniformity and Enhanced Stability
Source: Adv Sci (Weinh). 2023 Dec 19;11(10):2308153. doi: 10.1002/advs.202308153 (PMC10933648; doi:10.1002/advs.202308153)
Supplement: Supplementary file 1 — Supporting Information [file ADVS-11-2308153-s001.pdf]

ADVANCED SCIENCE
Open Access

Supporting Information

for *Adv. Sci.*, DOI 10.1002/advs.202308153

Ozone-Induced Rapid and Green Synthesis of Polydopamine Coatings with High Uniformity and Enhanced Stability

Liru Tan, Tang Zhu, Yuchan Huang, Huixin Yuan, Ludi Shi, Zijuan Zhu, Pingping Yao, Caizhen Zhu* and Jian Xu*

Supporting Information

Ozone-induced rapid and green synthesis of polydopamine coatings with high uniformity and enhanced stability

Liru Tan, Tang Zhu, Yuchan Huang, Huixin Yuan, Ludi Shi, Zijuan Zhu, Pingping Yao, Caizhen Zhu* , Jian Xu*

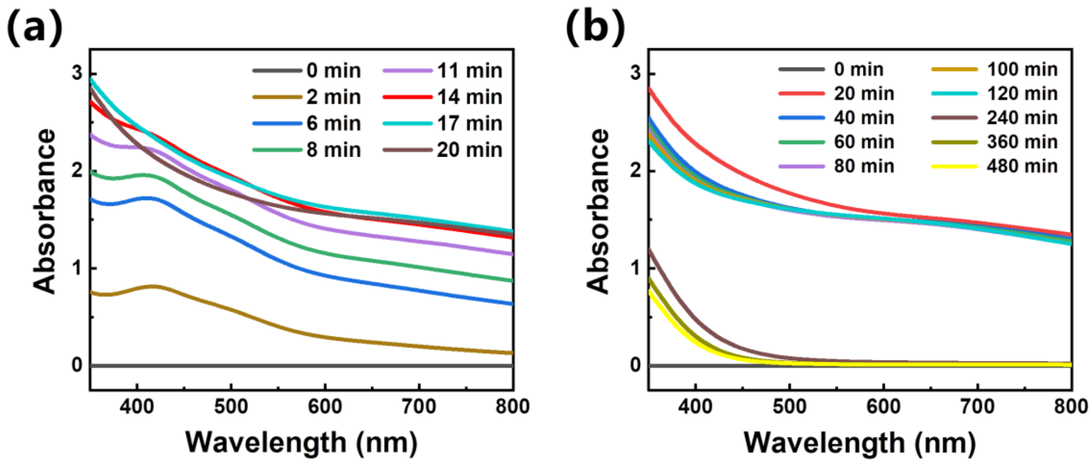

Figure S1. Time-dependent UV-vis absorbance of dopamine in Tris-HCl buffer solution (pH = 8.5) containing ozone: (a) 0~20 min and (b) 0~480 min.

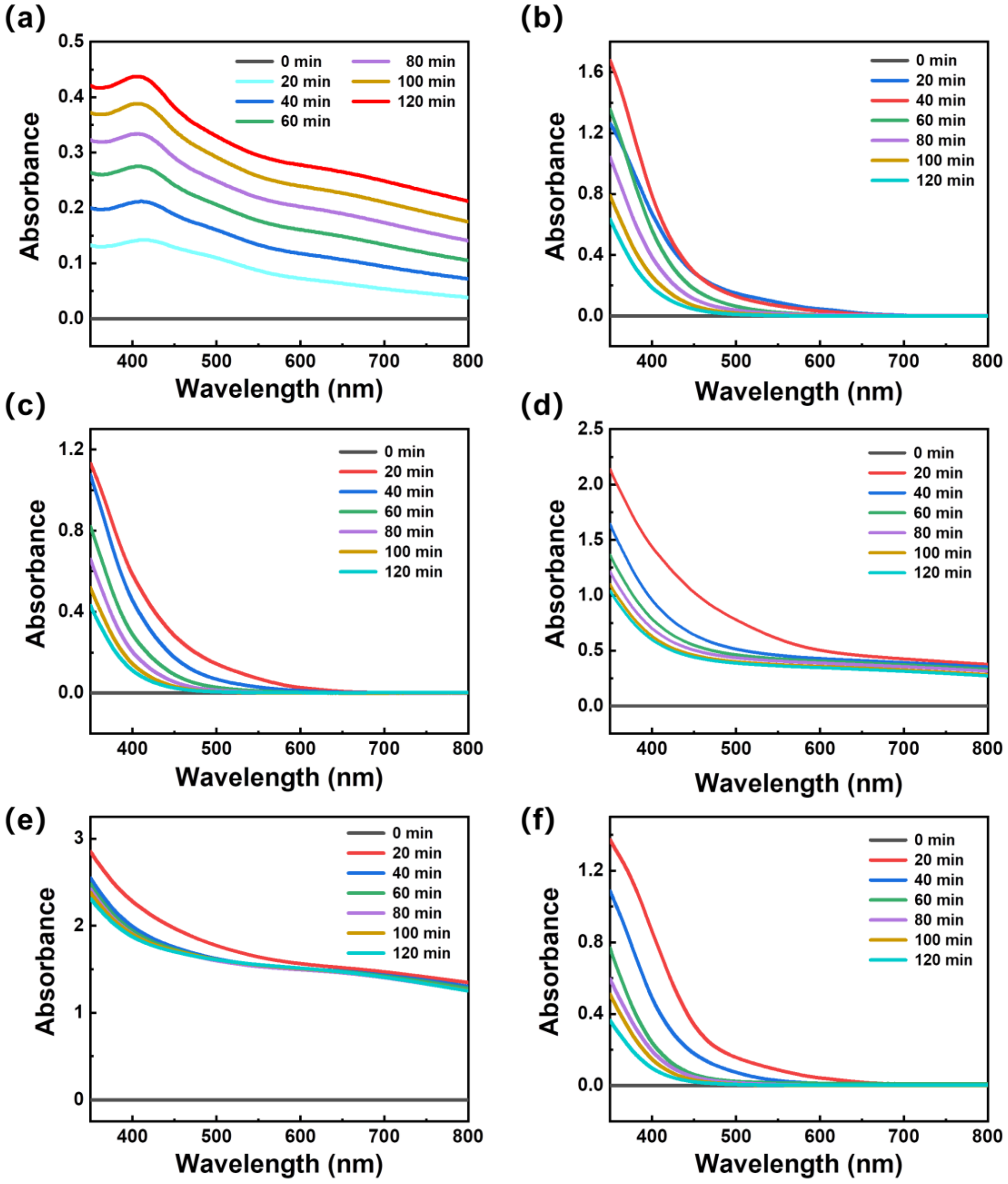

Figure S2. Time-dependent UV-vis absorbance of dopamine solution under different conditions: (a) air, pH = 8.5, (b) ozone, pH = 4.0, (c) ozone, pH = 5.0, (d) ozone, pH = 7.0, (e) ozone, pH = 8.5, (f) ozone, pH = 10.0.

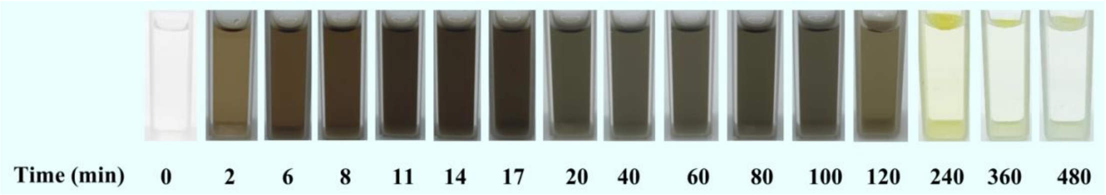

Figure S3. Color changes of dopamine solution (pH = 8.5) containing ozone with the running time.

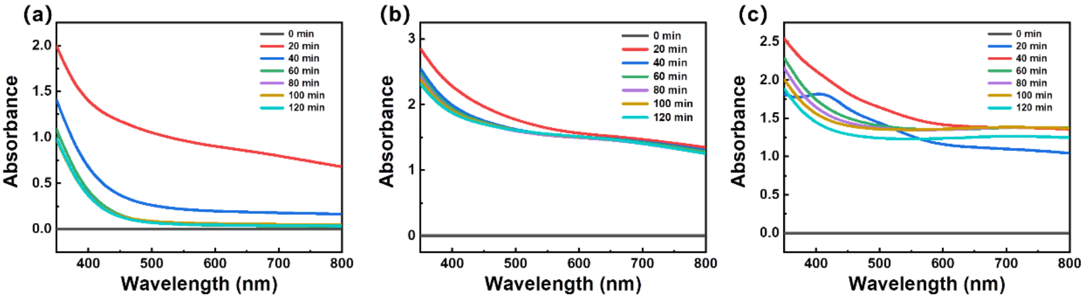

Figure S4. Time-dependent UV-vis absorbance of dopamine solution (pH = 8.5) with different ozone concentrations. Buffer volume: (a) 50 mL, (b) 100 mL, (c) 200 mL.

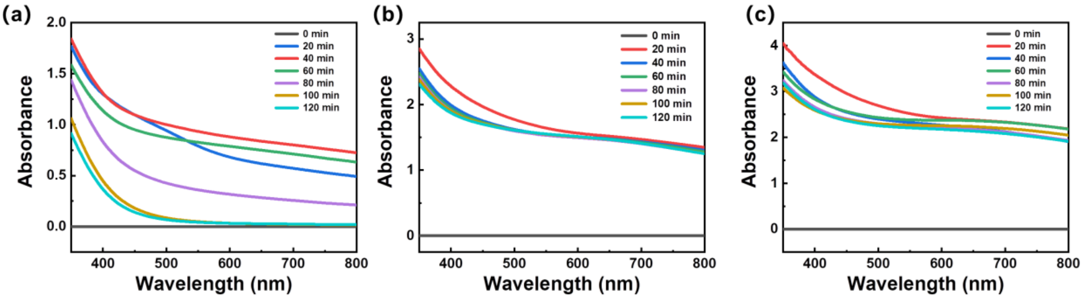

Figure S5. Time-dependent UV-vis absorbance of dopamine solution (pH = 8.5) at different temperatures: (a) 5 ℃, (b) 25 ℃ and (c) 60 ℃.

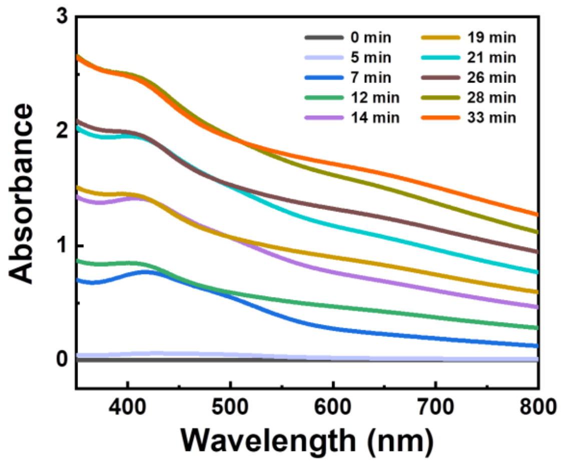

Figure S6. Time-dependent UV-vis absorbance of dopamine solution by turning on (2 min) and off (5 min) the ozone gas at pH = 8.5.

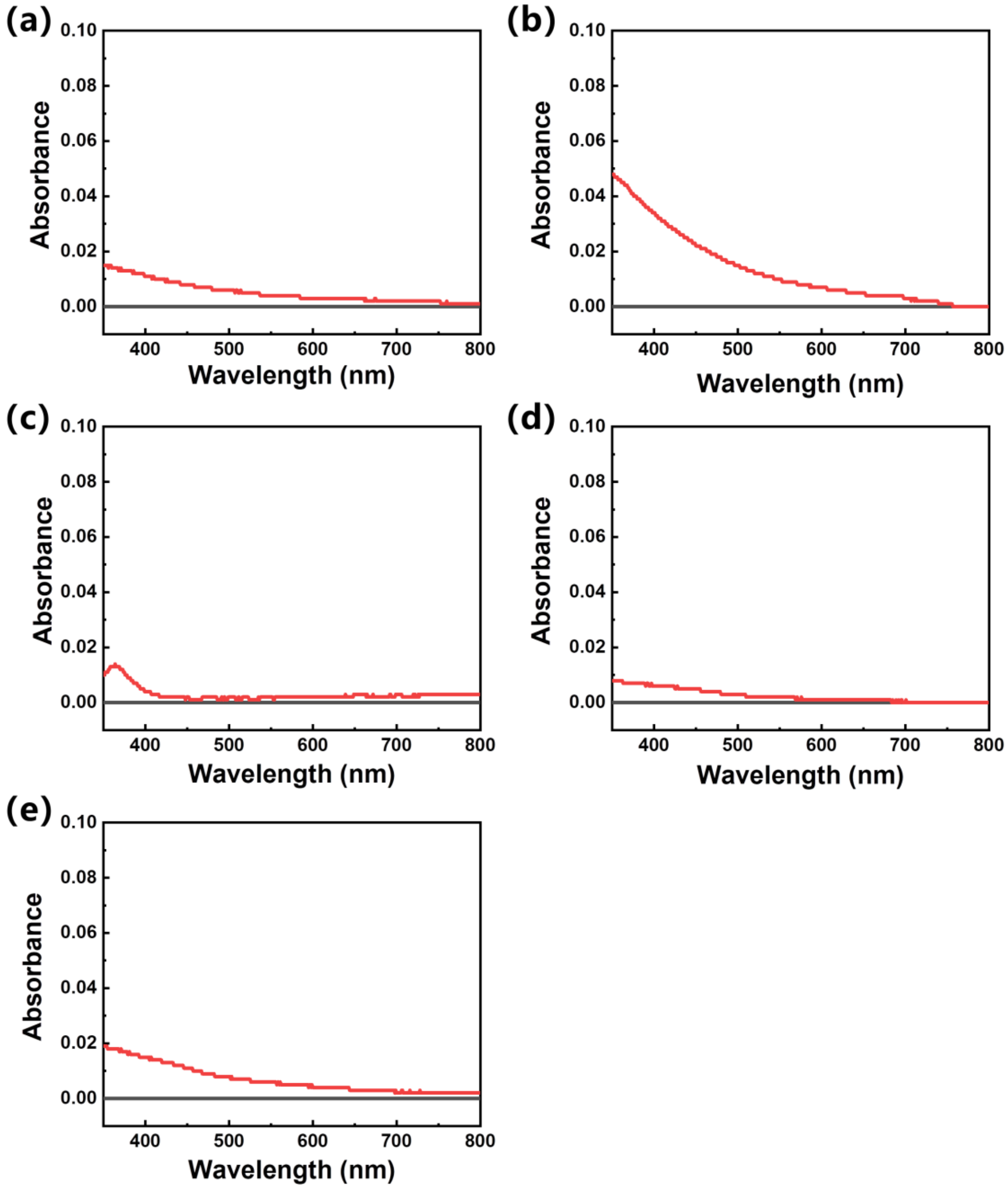

Figure S7. Silicon wafers coated with ozone-induced PDA were immersed in various solutions for 2 hours. UV-vis spectrum of the eluents: (a) 0.1 M HCl, (b) 0.1 M NaOH, (c) acetone, (d) DMF, (e) DMSO.

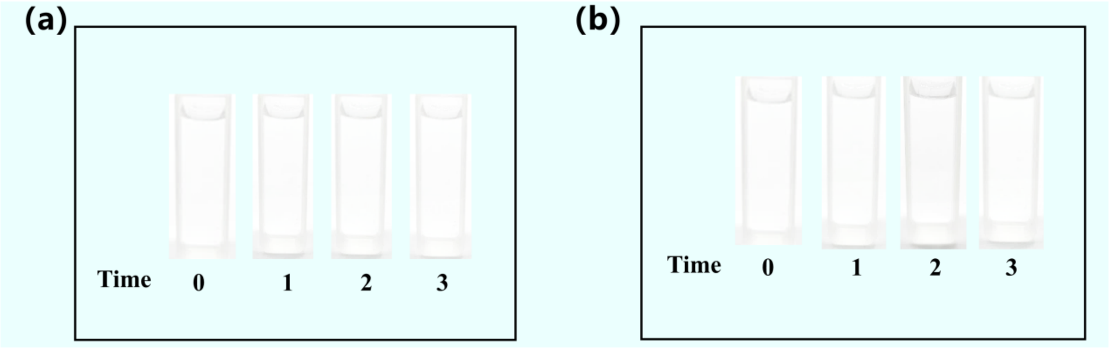

Figure S8. Digital photographs of the eluents in the mechanical stability tests: (a) ultrapure water, (b) ethanol.

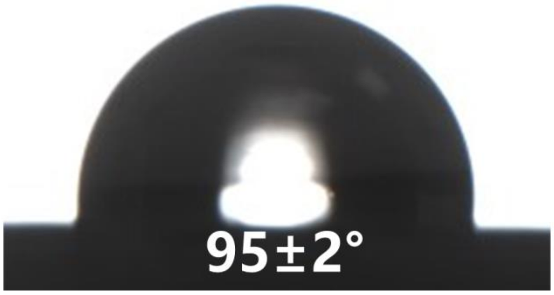

Figure S9. WCAs of the air-assisted PDA-coated PTFE after tape experiment.
